# Evaluating the potency of laser-activated antimicrobial photodynamic therapy utilizing methylene blue as a treatment approach for chronic periodontitis

**DOI:** 10.3389/froh.2024.1407201

**Published:** 2024-05-30

**Authors:** Manoj Kumar Karuppan Perumal, Remya Rajan Renuka, Prabhu Manickam Natarajan

**Affiliations:** ^1^ Center for Global Health Research, Saveetha Medical College and Hospitals, Saveetha Institute of Medical and Technical Sciences, Chennai, India; ^2^ Department of Clinical Sciences, College of Dentistry, Centre of Medical and Bio-Allied Health Sciences and Research, Ajman University, Ajman, United Arab Emirates

**Keywords:** chronic periodontitis, antimicrobial photodynamic therapy, low-level laser therapy, methylene blue, periodontal pathogens

## Abstract

Chronic periodontitis is a ubiquitous inflammatory disease in dental healthcare that is challenging to treat due to its impact on bone and tooth loss. Conventional mechanical debridement has been challenging in eliminating complex subgingival biofilms. Hence, adjunctive approaches like low-level laser antimicrobial photodynamic therapy (A-PDT) utilising methylene blue (MB) have been emerging approaches in recent times. This review evaluates the latest research on the use of MB-mediated A-PDT to decrease microbial count and enhance clinical results in chronic periodontitis. Studies have shown the interaction between laser light and MB generates a phototoxic effect thereby, eliminating pathogenic bacteria within periodontal pockets. Moreover, numerous clinical trials have shown that A-PDT using MB can reduce probing depths, improve clinical attachment levels, and decrease bleeding during probing in comparison to traditional treatment approaches. Notably, A-PDT shows superior antibiotic resistance compared to conventional antibiotic treatments. In conclusion, the A-PDT using MB shows promise as an adjunctive treatment for chronic periodontitis. Additional research is required to standardize treatment protocols and assess long-term outcomes of A-PDT with MB in the treatment of periodontitis.

## Introduction

Chronic periodontitis, an endemic inflammatory condition of the gums observed within the field of dental healthcare; poses an alarming challenge due to intricate involvement in bone and tooth loss, thus causing several diseases such as pneumonia, cancer, cardiovascular diseases, autoimmune diseases, etc (1). Chronic periodontitis has a significant spread in the worldwide population, with estimates ranging from 20% to 50%, indicating its widespread prevalence as a disease (2). The primary cause is the accumulation of complex polymicrobial biofilms in the subgingival pockets surrounding the teeth. These plaque colonies adhere strongly and provoke a chronic immunoinflammatory response, leading to the elimination of the connective tissue, periodontal ligament cementum and alveolar bone (3). Conventional approaches have relied largely on mechanical disruption and physical removal of accessible biofilms through scaling, root planing and surgery. The presence of bacteria in biofilms poses significant challenges to completely eradicating them, thereby leading to disease recurrence in a significant number of patients (4).

However, there has been a considerable surge in exploring adjunctive treatments, and one such innovative method gaining traction is low-level laser-activated photodynamic therapy utilizing methylene blue (MB) (5). Low-level laser treatment promotes the proliferation of adenosine triphosphate at the mitochondrial level (6). A laser emits a specific wavelength of light to increase reactive oxygen species (ROS) production through photosensitizer (PS). This treatment is considered a safe and impressive therapy since it has no long-term side effects (7). The dominant used PS in dentistry is Photofrin, but in recent times, MB has been considerably utilized as PS. Antimicrobial photodynamic therapy (A-PDT) uses light to activate MB, which produces ROS and damages bacterial cells (8). This process oxidizes various cell components, resulting in microbicidal effects against periodontal pathogens. A-PDT has multiple advantages, including its ability to suppress bactericidal action against a wide range of bacteria, penetrate biofilms, promote microbial cytotoxicity, enhance wound healing, and induce tissue remodelling (9, 10). The wavelengths at which MB absorbs most intensely are approximately 660 nm, which is within the red laser emission range. In other words, cheap diode lasers can be used to activate it. Additional research is required to develop MB-mediated A-PDT protocols that specifically focus on effective treatment outcomes and managing infection in various oral conditions such as chronic periodontitis (11).

Periodontal diseases are typically triggered by anaerobic bacteria like *Porphyromonas gingivalis (P.gingivalis), Actinobacillus actinomycetemcomitans (A. actinomycetemcomitans) and Tannerella forsythia (T. forsythia) P. gingivalis*, which is widely recognized as the primary causative agent in chronic periodontitis among adult individuals. The *A. actinomycetemcomitans* is commonly considered the primary pathogen in cases of aggressive periodontitis (12). The robust *in vitro* findings emphasise MB-mediated laser-activated A-PDT potent bactericidal efficacy against these anaerobic gram-negative bacteria even at short exposure times. Unlike antibodies, MB-mediated A-PDT has the competence to eliminate bacterial cells within intact biofilms. Hence, A-PDT techniques exhibit the promise of eliminating residual subgingival infection unreachable by mechanical instrumentation alone (13). Beyond bacterial cytotoxicity, another research on the anti-inflammatory effects of MB photoactivation suppresses inflammatory markers and mediators—including cytokines, chemokines, TNF-α, IL-1β, PGE2, and nitric oxide (14). Such immuno-modulatory properties can facilitate the resolution of inflammation in diseased periodontal tissues. Furthermore, studies have also emphasized A-PDT ability to improve clinical outcomes like reduced probing depths and bone loss *in vivo* (15). However, researchers have evaluated the clinical usefulness of MB as a complementary treatment to conventional mechanical debridement for managing chronic periodontitis in patients due to their accumulated understanding of MB multifactorial therapeutic effects. Initial systematic reviews of these clinical studies emphasize the significant potential benefits of reducing microbial load and improving clinical parameters over extended periods, with minimal adverse effects (16). Therefore, this review aims to analyze the existing evidence regarding laser-activated A-PDT using MB against periodontal pathogens, particularly those related to chronic periodontitis. It evaluates the mechanisms, existing research outcomes, and discusses key parameters to enhance the delivery of this emerging adjunct therapy, and its potential in combating chronic periodontitis.

## Fundamental mechanism underlying the photodynamic therapy

**Figure 1:**
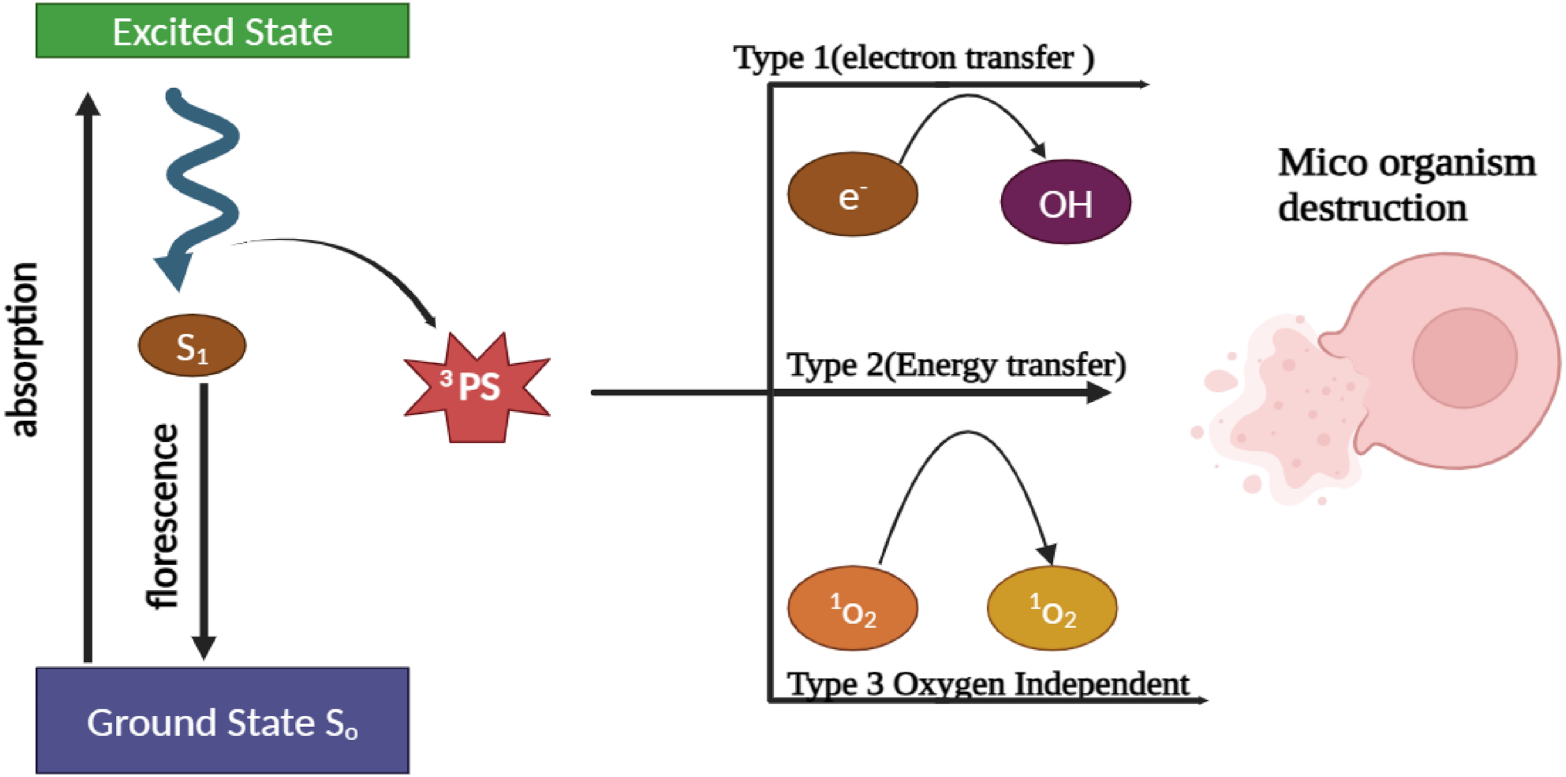
Schematic illustration depicting the mechanism of bacterial cell wall destruction through the use of A-PDT therapy.

A PS, a light source, and oxygen in the tissue constitute the basic elements of PDT. PS is a nontoxic dye that can be selectively absorbed and retained in specific cells. The common PS used during PDT treatment are porphyrins, chlorins, dyes such as MB and toluidine. PDT is based on the activation of PS upon exposure to a specific wavelength of light. This activation involves transitioning the PS from a low-energy ground state (S0) to an excited singlet state (S1). The S1 state is transient and not very stable but can undergo intersystem crossing to become a more stable and longer-lasting excited triplet state. In order to retain stability, the S1 state can revert back to the ground state through fluorescence or internal conversion, which results in the loss of energy. Alternatively, it can also transform into an excited triplet state (S3) by altering the electron spin. The S3 state initiates two pathways influenced by oxygen. These pathways involve the interaction of the triplet state (T) with oxygen in the surrounding tissue, leading to the production of highly ROS like singlet oxygen and free radicals. The process is depicted in Figure 1 (17–19). Molecules in the T state have the capability of emitting light, which is known as phosphorescence. This occurs either by returning to ground states or by undergoing additional reactions through type 1 and type 2 pathways. Radical ions, such as superoxide, hydroxyl, and lipid-derived radicals, are produced through electron transfer from PS to substrate in the type 1 pathway. Furthermore, the type 2 pathways involve the transfer of energy from the PS triplet to the ground state, resulting in the production of singlet oxygen and the oxidation of various biomolecules such as proteins, nucleic acids, or lipids. This leads to the production of cytotoxic effects (20–22).

## Optimization of laser-activated A-PDT treatment

The key factor involved in determining the outcomes of A-PDT is defining optimal activation protocols that provide a targeted bactericidal effect while minimizing damage to surrounding tissues. Thus, the laser and PS have become significant factors for A-PDT therapy. In the case of MB-mediated A-PDT, the maximum adsorption peak was observed at 660–670 nm using low-level red lasers. Meanwhile, the diode lasers provide high-intensity monochromatic light with precision fibre-optic delivery; allowing controlled irradiation adapted to periodontal pocket topography (22). The diode family, Er,Cr:YSGG, and Nd:YAG lasers (635 nm – 2,780 nm) effectively remove debris and bacteria from root canals, using optical fibers (200 μm – 200 mm), power output (40 mW – 1.5 W), and irradiation duration (140 μs – 3 mins). For photodynamic therapy, methylene blue and toluidine blue (1–5 mins application) are utilized. Diode lasers (810 or 980 nm) with anti-bactericidal properties can reach the periapical region (23). Research on A-PDT utilizing EmunDo and diode laser against the dental pathogen *Lactobacillus acidophilus*, which affects the cavity. The results showed that by using the EmunDo and Diode laser systems, a considerable reduction in colonies was achieved (24). In addition to considering laser parameters, the A-PDT is also affected by other important factors including power density (irradiance), exposure time per site, and beam mode/diameter. These variables collectively contribute to the energy density (radiant exposure), which plays a crucial role in determining the phototoxic effect and overall treatment success. Toluidine blue O (TBO) activated by red LED light was found to inhibit cariogenic biofilms in an *in vitro* study. Biofilm analysis and cytotoxicity tests were used to identify the ideal parameters. It was found that while TBO at concentrations of 50 and 100 μg/ml preserved cell viability, higher concentrations prevented the formation of biofilms. The study stresses the importance of optimizing TBO concentration and light energy for better biofilm elimination (25). Likewise, the study involved 30 subjects: 15 patients received retreatment of the root canal in combination with 980 nm diode laser irradiation and 15 were given placebos. For each sample in a pulsing mode, the power output shall be 20 s at 1.5 W and 100 Hz. The diode laser significantly improved healing at 3 to 12 months follow-up (*P* < 0.05) and had 45% more healed cases than the placebo (26).

## Current and alternative treatments for chronic periodontitis

The conventional treatment for chronic periodontitis implies mechanical disruption and eliminating the bacterial biofilms, through scaling and root planing. This non-surgical treatment utilizes numerous specialized instruments to mechanically debride plaque and calculus deposits from the gum line. As an adjunct, antimicrobial mouth rinses may be prescribed. However, limitations in eliminating complex residual biofilms can drive recolonization (27). General antibiotics such as amoxicillin and metronidazole in combination with Scale root planing (SRP) have been extensively evaluated to improve microbial control; long-term usage of these antibiotics risks antibiotic resistance in non-target commensal bacteria. The potential adjuvant interventions like controlled-release vehicles, antibiotics embedded in bone grafts, PS dyes, host modulatory agents, probiotics and laser/phototherapy have been investigated for minimized spread of antimicrobial-resistant genes. Nevertheless, each approach has its inherent limitations and currently restricts widespread adoption (9).

Non-pharmacological adjuncts like ultrasonic devices, ozone therapy, and air-polishing with glycine powder show some improvement but lack strong clinical evidence (28). A study on the treatment of type 2 diabetes (T2D) and periodontitis discovered that a combination of antibiotics and SRP was successful in reducing HbA1c levels and decreasing probing pocket depth (PPD) after 3 months. However, after 6 months, only SRP was found to effectively lower HbA1c levels. Both SRP and antibiotics showed significant reductions in PPD. As a result, it is advisable to consistently utilize SRP for patients with T2D and periodontitis, as systemic antibiotics only provide temporary benefits (29). A study conducted on 58 patients with chronic periodontitis revealed that all groups, which underwent various treatments, demonstrated significant reductions in probing depth, clinical attachment loss, and bleeding on probing after 6 and 12 weeks. Furthermore, the addition of PDT treatment to scaling and root planing resulted in a significant decrease in bleeding in 5 mm probing depth pockets after 12 weeks (30). Similarly, another study was conducted to investigate the effectiveness of additional treatment methods for apical periodontitis in permanent teeth. The study evaluated several treatments, such as A-PDT, laser canal irradiation, ozone therapy, and ultrasonic irrigation. The results indicated that there was no statistically significant difference in healing rates or pain prevalence between the group receiving adjunctive treatment and the control group (31). In conclusion, the current guidelines still recommend SRP as the primary method of mechanical debridement. The use of additional measures, such as locally delivered antimicrobials, should only be considered for specific cases in which non-responsive sites pose significant treatment challenges. Therefore, there is a need to develop novel strategies that target biofilms specifically, offering improved and long-lasting effectiveness as well as safety advantages.

## Non-invasive A-PDT therapy for the treatment of periodontitis: an overview

A-PDT utilizes lasers and PS agents to eliminate bacteria associated with periodontitis. It is extremely effective in eradicating bacteria, while also having minimal toxicity and being cost-effective. Various PS, including MB, toluidine blue, indocyanine green, malachite green, erythrosine dyes, rose bengal, the radiochlorine group, and curcumin, may be safely used with different light sources without causing any harm to the patient (32). A study was conducted to investigate the effects of A-PDT in patients who were diagnosed with T2D and periodontitis. The results, observed at 90 and 180 days, showed a significant reduction in pocket depth, bleeding on probing, and the number of remaining pockets (33). Researchers compared A-PDT and antibiotics with non-surgical treatment for aggressive periodontitis. The results indicate that the use of both A-PDT and antibiotics as adjuncts is effective. Both approaches also showed long-term improvements in periodontal parameters (34). The research was conducted to test the potency of A-PDT when combined with laser application against a specific periodontal pathogen, *A. actinomycetemcomitans*. The findings of the study indicated that using a wavelength of 940 nm, along with PS-dimethyl phenothiazine chloride, and irradiating with a diode laser for 30 s at a power of 5 W, resulted in a significant reduction of *A. actinomycetemcomitans in vitro* (35). Thirty-six patients with periodontal disease were randomly assigned to either the intervention or control group. Prior to treatment, all participants underwent scaling and root planning. Evaluations were conducted at three intervals, which included microbiological and clinical examinations. Laser therapy was administered using two PS solutions. The results of the study indicated that there were no significant differences between the groups in terms of reduction in bacteria levels and testing depths (36).

## Properties favouring methylene blue as an A-PDT photosensitizer

MB is a hydrophilic phenothiazinium cationic dye that has the ability to easily penetrate bacterial cell walls. MB-mediated antimicrobial A-PDT boosts neutrophil adhesion while inhibiting phagocytic activity (37). It possesses the ideal combination of a high quantum yield and excellent chemical stability necessary for effective photodynamic action. The positive charge of MB enhances its ability to adhere to negatively charged microbial cell surfaces and facilitates specific uptake and photosensitization (38). In addition to its direct antimicrobial properties, research indicates that the activation of MB via A-PDT can also trigger secondary local responses that are dependent on oxygen. These responses are beneficial for the healing of periodontal tissues and supporting the immune system (39). The absorption spectrum of MB exhibits a peak at approximately 660 nm, aligning with the emission range of affordable diode lasers. This alignment enables sufficient tissue penetration and photodynamic activation within periodontal pockets using cost-effective and small-sized light sources. Consequently, this ease of clinical implementation enables the effective utilization of this approach. Using these PS, A-PDT can reduce the number of harmful bacteria in periodontitis. It can also be combined with other treatments (40).

The various laser wavelengths and PS for A-PDT treatments are mentioned in Table 1. The effects of A-PDT therapy on patients with grade C periodontitis were examined in this study. Two groups of sixty-four teeth, with one receiving A-PDT therapy during SRP and the other receiving a placebo. In comparison to the control group, the three-month results for the A-PDT therapy group indicated a reduction in probing depth and an increase in clinical attachment. These results imply that A-PDT therapy might be helpful in the management of periodontitis (50). Similarly, A-PDT therapy improves clinical outcomes for people with T2D, according to an analysis of 11 studies. The study showed that when compared to receiving standard periodontal treatment, using A-PDT therapy led to more notable reductions in bleeding on probing and probing depth at 3 and 6 months (51).

**Table 1:** The parameters of laser irradiation and the outcomes observed in antimicrobial photodynamic therapy (A-PDT).

Author	Light source and wavelength	PS	Main outcomes	Research
Mocanu et al. (41)	Laser light: 660 nm	Phenothiazine chloride	Results indicated that PDT as an adjunctive therapy significantly improved clinical parameters and reduced microbiological load after one month. These improvements were sustained at the six-month follow-up, particularly in patients with severe periodontitis.	*In vivo*
Cosgarea et al. (42)	Laser light: 660 nm	HELBO blue	The study found significant clinical improvements in periodontal pockets up to 12 months with LDD/PDT combined with subgingival instrumentation	*In vivo*
Baeshen et al. (43)	Diode laser: 670 nm	Methylene blue	The study compared the effectiveness of PDT on adolescent orthodontic patients with gingivitis. Both groups showed improved plaque scores and bleeding, with Group B starting with lower bacterial counts. Pain scores were similar between groups. Reductions in IL-6 and TNF-α levels were seen in both groups at different times. PDT proved effective in reducing periodontal microbial load in these patients.	*In vivo*
Alrabiah et al. (44)	670 nm diode laser	Methylene blue	A randomized clinical trial found that photodynamic inactivation (PDI) and nystatin (NST) are both effective in reducing Candida colony counts on dentures and palates in patients with denture stomatitis (DS). PDI was more effective than NST in reducing Candida counts on dentures, but both treatments were equally effective in reducing Candida prevalence.	*In vivo*
Alvarenga et al. (45)	Red laser: 660 nm	Methylene blue	A study of 30 patients showed that MB in a surfactant vehicle, irradiated for 5 min at 660 nm, achieved significant microbial reduction, unlike aqueous methylene blue. Irradiation methods & photosensitizer stability are crucial for successful clinical A-PDT.	*In vivo*
Cadore et al. (46)	Diode laser: 660 nm	Phenothiazine chloride	A clinical trial found that A-PDT plus surgical periodontal treatment improved probing depth and clinical attachment level in patients with severe chronic periodontitis. However, both groups had similar changes in subgingival microbiota, and the test group had more bacteria associated with periodontal disease at the end of the study.	*In vivo*
Skalerič et al. (34)	Laser light: 670 nm	HELBO Thera Lite laser, HELBO blue photosensitizer,	The study aimed to evaluate the efficacy of A-PDT and antibiotic therapy as adjuncts to non-surgical periodontal treatment for aggressive periodontitis. The findings revealed that both A-PDT and antibiotic therapy exhibited significant improvements in clinical parameters, including probing pocket depths, clinical attachment levels, and bleeding on probing, over a 12-month follow-up period. These results suggest that the adjunctive use of A-PDT or antibiotic therapy can enhance long-term periodontal outcomes and contribute to the effective management of aggressive periodontitis when combined with non-surgical periodontal therapy.	*In vivo*
Hokari et al. (47)	Diode laser: 670 nm	Methylene blue,	A study compared the effectiveness of A-PDT and minocycline ointment (MO) on chronic periodontitis patients. MO showed significant improvements in clinical parameters, bacterial counts, and cytokine levels, while A-PDT only affected clinical parameters. MO demonstrated positive effects on clinical, microbiological, and crevicular cytokine levels in periodontal pockets, whereas A-PDT did not show any significant effects.	*In vivo*
Hill et al. (48)	Diode laser: 808 nm	Indocyanine green	A clinical study of 20 patients found that ICG-based A-PDT did not significantly improve chronic periodontitis treatment outcomes compared with scaling and root planing alone, except for a temporary decrease in sulcus fluid flow rate in the A-PDT group	*In vivo*
Soundarajan et al. (49)	660 nm diode	Methylene blue	Two adjunctive therapies, A-PDT and Er, Cr: YSGG laser, were compared for improving periodontal health parameters. 36 subjects received SRP alone in one quadrant, SRP + Er, Cr: YSGG laser in another quadrant, and SRP + A-PDT in a different quadrant. Both adjunctive therapies significantly improved PI, GI, PD, and CAL compared to baseline. Er, Cr: YSGG laser showed better outcomes than a-PDT. Both adjunctive therapies were more effective than SRP alone in reducing periodontal parameters.	*In vivo*

## Conclusion

Chronic periodontitis poses a major threat to global health due to its involvement in systemic diseases and tooth loss pathogenesis. Complex residual biofilms are beyond the scope of conventional mechanical debridement techniques. This has led to the investigation of novel adjunctive strategies, including A-PDT, which uses MB as a PS. To evaluate the bactericidal efficacy of MB-mediated laser-activated A-PDT against important anaerobic periodontal pathogens. Additionally, research has revealed that when MB is activated, cytotoxic ROS are produced. These species enter biofilms and destroy bacteria, as well as trigger favourable immunomodulatory and wound-healing reactions. Based on clinical trials, this adjunctive approach offers numerous advantages including reduced bleeding and probing depths, as well as increased levels of attachment. A-PDT is a potential intervention with multiple benefits including its low cost, safety profile, and ease of use when combined with inexpensive red lasers. Moreover, future research should aim to refine treatment protocols, investigate complementary therapies, and conduct randomized controlled trials to confirm the effectiveness of MB-mediated A-PDT therapy for chronic periodontitis.
